# *Bacillus licheniformis*-based intensive fermentation of Tibetan tea improved its bioactive compounds and reinforced the intestinal barrier in mice

**DOI:** 10.3389/fmicb.2024.1376757

**Published:** 2024-06-12

**Authors:** Hui Zhu, Xiaoli Zhou, Caihong Shen, Zonghua Ao, Xiaonian Cao, Chuan Song, Muhammad Aamer Mehmood, Tao Wu, Jie Mei, Manli He, Yi Ma, Ning Wang

**Affiliations:** ^1^College of Bioengineering, Sichuan University of Science and Engineering, Zigong, China; ^2^Sichuan Province Engineering Technology Research Center of Liquor-Making Grains, Yibin, China; ^3^Luzhou Laojiao Co. Ltd., Luzhou, China; ^4^Department of Bioinformatics and Biotechnology, Government College University Faisalabad, Faisalabad, Pakistan; ^5^School of Food and Biological Engineering, Xihua University, Chengdu, China; ^6^Sichuan Jixiang Tea Co., Ltd., Ya'an, China; ^7^Laboratory Animal Center, Southwest Medical University, Luzhou, China

**Keywords:** Tibetan tea, *Bacillus licheniformis*, fermentation, metabolic analysis, intestinal barrier

## Abstract

Tibetan tea changes during microorganism fermentation. Research on microorganisms in Tibetan tea has focused on their identification, while studies on the influence of specific microorganisms on the components and health functions of Tibetan tea are lacking. *Bacillus licheniformis* was inoculated into Tibetan tea for intensive fermentation, and the components of *B. licheniformis*-fermented tea (BLT) were detected by liquid chromatography with tandem mass spectrometry (UHPLC-TOF-MS), and then the effects of BLT on intestinal probiotic functions were investigated by experiments on mice. The results revealed the metabolites of BLT include polyphenols, alkaloids, terpenoids, amino acids, and lipids. Intensified fermentation also improved the antioxidant capacity *in vivo* and the protective effect on the intestinal barrier of Tibetan tea. In addition, the enhanced fermentation of Tibetan tea exerted intestinal probiotic effects by modulating the relative abundance of short-chain fatty acid-producing bacteria in the intestinal flora. Therefore, intensive fermentation with *B. licheniformis* can improve the health benefits of Tibetan tea.

## 1 Introduction

Tibetan tea is a typical dark tea with a history of over 1,300 years. It is produced by post-fermentation, distinguishing it from other teas in terms of its sensory quality. Tibetan tea is dark brown in appearance, orange–red in color when prepared, and has a unique aroma. It tastes refreshing and sweet rather than bitter or astringent (Zhu et al., 2020; Zhou et al., 2022). Tibetan tea is generally processed in four steps: fixation (kill-green), kneading, pile-fermentation, and drying, of which pile-fermentation is the most crucial step. During pile-fermentation, microorganisms grow alternately and produce extracellular enzymes under high-temperature and high-moisture environments, which change the biochemical composition of the tea by inducing various biochemical reactions and metabolic pathways and producing new compounds, resulting in the unique sensory characteristics and health functions of the tea (Li et al., 2018; Zheng et al., 2020; Liu et al., 2022). Consequently, microorganisms have a vital influence on the quality of Tibetan teas. Tibetan tea contains various bioactive substances, including tea polyphenols, tea polysaccharides, and theabrownin, leading to various health benefits associated with its consumption, such as antioxidant, lipid-lowering, immunomodulatory, and antihypertensive effects (Chen et al., 2018; Zheng et al., 2020; Zhu et al., 2020).

Numerous studies have demonstrated the beneficial effects of tea on the intestinal flora (Liu et al., 2018, 2020; Bond and Derbyshire, 2019; Zhou et al., 2021). The gut microbiota comprises ~10^14^ microorganisms in the human gut, which is an indispensable metabolic and endocrine organ acquired by human beings. The gut microbiota can produce rich metabolites, such as short-chain fatty acids (SCFAs), which have important effects on the human immune and metabolic systems (Rowland et al., 2018; Adak and Khan, 2019). Tibetan tea and its functional ingredients can regulate intestinal flora and maintain intestinal health by promoting the growth of beneficial bacteria, inhibiting pathogenic bacteria, and regulating the content of intestinal flora metabolites (Samynathan et al., 2023; Tan et al., 2023).

Furthermore, microbial fermentation can enhance the biological characteristics of raw materials and improve the beneficial health benefits of natural plant products through biotransformation. Using probiotics to ferment plants can improve the edible value and flavor of raw materials and enhance their beneficial characteristics (Yong et al., 2019; Park et al., 2023). The pile-fermentation process of Tibetan tea is characterized by high-temperature solid-state fermentation, during which thermophilic microorganisms promote the transformation of compounds in the tea leaves (Li et al., 2018). *Bacillus licheniformis* is the dominant bacterium involved in the fermentation of Tibetan tea. It is a probiotic bacterium with high-temperature tolerance; it can grow in a high-temperature environment and possesses phytase, protease, cellulase, and xylanase activities (Qi et al., 2023).

This study isolated and purified *B. licheniformis* from Tibetan tea and aimed to investigate the effect of intensive fermentation caused by *B. licheniformis* on the chemical composition and bioactive functions of Tibetan tea. This study provides a theoretical basis for the development of Tibetan tea with health-improving functions.

## 2 Materials and methods

### 2.1 Preparation of *B. licheniformis* bacterial suspension and fermentation of Tibetan tea

The bacterial strain CX2 was previously isolated and identified from Tibetan tea. A phylogenetic tree, shown in Supplementary Figure S1 and constructed using MEGA 11, revealed a 99.54% sequence similarity between strain CX2 and KP743132.1 *Bacillus licheniformis* strain YM6. Both strains clustered together on the same branch, identifying strain CX2 as *B. licheniformis*.

*B. licheniformis* was inoculated in LB Broth and cultured at 37°C with a shaking speed of 180 rpm for 24 h. The bacterial count was determined, and the culture was diluted with normal saline to achieve a concentration of 10^3^ CFU/mL. Subsequently, 1% of the bacterial suspension was inoculated into a tea culture medium and incubated at 37°C with a shaking speed of 180 rpm for 4 days. Among them, the tea culture medium was prepared by extracting 100 g Tibetan tea with 2 L distilled water at 100°C for 30 min. The mixture was then filtered through four layers of gauze and sterilized at 121°C for 20 min.

Tibetan tea (15 g) was placed in a 100 mL conical flask and 1 mL of the bacterial suspension was added, while 1 mL of distilled water was added to the control group. They were mixed evenly and processed for intensive fermentation at 37°C for 2 days, 50°C for days 3 and 4, and 65°C for days 5–7.

### 2.2 Preparation of Tibetan tea extracts

First, 10 g Tibetan tea (including Tibetan tea after enhanced fermentation by *B. licheniformis* and non-fermented Tibetan tea) was ground and passed through an 80-mesh sieve. Extraction was carried out in 100°C water (1:10, *w/v*) for 30 min with gentle stirring, and repeated extractions. Subsequently, all aqueous extracts were combined and centrifuged for 10 min at 1,760 × g. The filtrate was concentrated with a rotary evaporator and freeze-dried after being frozen at −80°C for 12 h. Finally, the resulting sample was stored at −80°C for subsequent analyses.

### 2.3 Untargeted metabolomics

The chemical compositions of non-fermented and fermented Tibetan teas were determined using liquid chromatography with tandem mass spectrometry (LC–MS/MS). All tea samples were injected into a UHPLC–TOF–MS system (Shimadzu, Tokyo, Japan). The detailed instrumentation and procedures used here were described previously (Wang et al., 2023). Untargeted metabolomics analysis was performed using SCIEX OS 2.0. The data were fitted using principal component analysis (PCA) to identify the overall distribution of the samples. The orthogonal partial least squares-discriminant analysis (OPLS-DA) model was established based on PCA for differential metabolite studies of the samples, and the results were plotted as heat maps using the OmicStudio tools at https://www.omicstudio.cn.

### 2.4 Mouse model experiment

Male C57BL/6 mice (18–22 g) aged 6 weeks and in good health were obtained from SPF Biotechnology Co., Ltd. (Beijing, China). The animal experiments were conducted strictly following the guidelines of SPF Biotechnology Co., Ltd. and were approved by the Laboratory Animal Ethics Committee (approval number: swmu20220137).

All mice were housed in the cage lined with dry shavings and maintained in the standard environment (temperature, 22–25°C; humidity, 40–60%; 12/12 h light/dark cycle) with free access to food and water. After a week of adaptive feeding, 18 six-week-old SPF male mice were randomly divided into three groups, with six in each group: control group, fed 0.2 mL of double distilled water (C); non-fermented Tibetan tea group, fed 0.2 mL of non-fermented Tibetan tea (TT); and tea fermented using *B. licheniformis* group, fed 0.2 mL of Tibetan tea that was intensively fermented using *B. licheniformis* (BLT). The prepared dry powder was dissolved in water to obtain a 12 mg/mL Tibetan tea solution, which was administered daily via intragastric gavage. Treatment was administered for 2 weeks. After the treatment period, the mice were fasted overnight and sacrificed by decapitation. The liver and colon tissues, and colon contents were quickly collected aseptically. All samples were stored at −80°C until further analyses.

### 2.5 Measurements of antioxidant capacity *in vivo*

The liver tissue was rinsed with ice-cold saline and weighed. The sample was then subjected to homogenization with phosphate-buffered saline buffer (pH 7.4) to obtain 10% (*w/v*) of the sample to be tested. The content of antioxidant enzymes including T-AOC, GSH, and CAT in the liver were detected using the commercial assay kits (Solarbio, Beijing, China) following the pre-set standard protocols.

### 2.6 Histological assays

The colon tissue was promptly immersed in a 4% polyformaldehyde solution for fixation. Subsequently, the tissues were dehydrated, embedded in paraffin, and sectioned. The colon tissue sections were stained with hematoxylin–eosin (H&E). Furthermore, Alcian Blue–periodic acid Schiff (AB–PAS) staining was used to label goblet cells in the colon sections. The resulting sections were observed under a light microscope equipped with a camera.

### 2.7 qRT-PCR analysis

The liver and colon tissues were used to extract total RNA using the FastPure^®^ Cell/Tissue Total RNA Isolation Kit (RC112, Vazyme Biotech Co., Ltd, Nanjing, China) according to the manufacturer's instructions. The RNA samples were assessed for A260/A280 values using a NanoDrop One Ultra-Micro Spectrophotometer (Thermo Scientific, USA), aiming for 1.8–2.0. For cDNA synthesis, the Hiscript^®^ III RT SuperMix for qPCR (+gDNA wiper) (Vazyme Biotech Co., Ltd., Nanjing, China) was employed. Taq Pro Universal SYBR qPCR Master Mix (Vazyme Biotech Co., Ltd., Nanjing, China) was used to quantify gene expression; the specific primer sequences are presented in Table 1. The 2^−Δ*ΔCt*^ method was employed for data analysis, with the β-actin gene (*Actb*) serving as the internal control.

**Table 1:** RT-qPCR primer sequences.

**Target**	**Sequence (5^′^-3^′^)**
*GSH*	F: CCACCGTGTATGCCTTCTCC
	R: AGAGAGACGCGACATTCTCAAT
*Cu/Zn-SOD*	F: AACCAGTTGTGTTGTCAGGAC
	R: CCACCATGTTTCTTAGAGTGAGG
*Iκ*Bα**	F: TGAAGGACGAGGAGTACGAGC
	R: TGCAGGAACGAGTCTCCGT
*iNOS*	F: GTTCTCAGCCCAACAATACAAGA
	R: GTGGACGGGTCGATGTCAC
*COX-2*	F: GGTGCCTGGTCTGATGATG
	R: TGCTGGTTTGGAATAGTTGCT
*NF-κB*	F: CAATGGCTACACAGGACCA
	R: CACTGTCACCTGGAACCAGA
*Occludin*	F: AAGCAAGTGAAGGGATCTGC
	R: GGGGTTATGGTCCAAAGTCA
*Claudin-1*	F: GCTGGGTTTCATCCTGGCTTCT
	R: CCTGAGCGGTCACGATGTTGTC
*ZO-1*	F: TCATCCCAAATAAGAACAGAGC
	R: GAAGAACAACCCTTTCATAAGC
*ZO-2*	F: GCTTTGGTGTGGACCAAGAT
	R: TCCATTATGGGTTTGCATGA
*Mucin-1*	F: TGGATTGTTTCTGCAGATTTT
	R: CCTGACCTGAACTTGATGCT
*Mucin-2*	F: CCCAGAAGGGACTGTGTATG
	R: TGCAGACACACTGCTCACA
*TNF-α*	F: GCATGGTGGTGGTTGTTTCTGACGAT
	R: GCTTCTGTTGGACACCTGGAGACA
*β-actin*	F: ACCTCCAGGACGACGACTTTGAT
	R: GTGTCTTCTGCACGTACTCCA

### 2.8 DNA extraction and amplicon sequencing of gut microbiota

Fresh and sterile intestinal contents were collected from each group of the mice, and genomic DNA extraction was conducted using the E.Z.N.A.^®^ Stool DNA Kit (Omega Bio-Tek, GA, USA). Subsequently, the concentration and purity of the DNA samples were determined using 1% agarose gel electrophoresis. The DNA samples were then placed in a centrifuge tube and diluted to 1 ng/mL in sterile water. Taking the diluted genomic DNA as the template, specific primers (515F and 806R) with Barcode and PHUSION^®^ High-Fidelity PCR Master Mix with GC Buffer (New England Biolabs) were used to amplify the specific regions. The samples were then mixed equivalently according to the concentration of the PCR products, and detection was conducted using 2% agarose gel electrophoresis. The target bands were recovered using an adhesive recovery kit (QIAGEN, Hilden, Germany).

The library was built using the NEBNext^®^ Ultra™ IIDNA Library Prep Kit, and the completed library was subjected to Qubit and Q-PCR quantification. After the library was qualified, NovaSeq6000 was used for up-sequencing.

### 2.9 Bioinformatics analyses based on the high throughput sequences of the gut microbiota

The reads of the samples were spliced using FLASH (V1.2.11, http://ccb.jhu.edu/software/FLASH/) to obtain Raw Tags. The Raw Tags were then quality-controlled using Fastp software to obtain high-quality Clean Tags. The Clean Tags were checked against the database using Usearch software (V10, http://www.drive5.com/usearch/) to detect chimeras and remove them to obtain the final validated data, the Effective Tags. For the Effective Tags obtained above, the DADA2 module in the QIIME2 software was used, and sequences with an abundance of less than five were filtered out to obtain the final Amplicon Sequence Variants (ASVs) and feature tables. Then, the classify-sklearn module in the QIIME2 software was used to compare the ASVs and databases to obtain the species information of each ASV. QIIME2 software was used to calculate the Shannon, Simpson, and Pielou_e indices. For beta diversity, species with statistically significant differences between groups were analyzed using the linear discriminant analysis (LDA) effect size (LEfSe).

### 2.10 Statistical analyses

The statistical analyses were performed using GraphPad Prism 9 (GraphPad Software, San Diego, CA, USA). All experimental data are expressed as mean ± standard error. Significance is presented as ^*^p < 0.05, ^**^p < 0.01, ^***^p < 0.001, and ^****^p < 0.0001.

## 3 Results

### 3.1 Intensive fermentation using *B. licheniformis* improved the chemical composition of Tibetan tea

**Figure 1:**
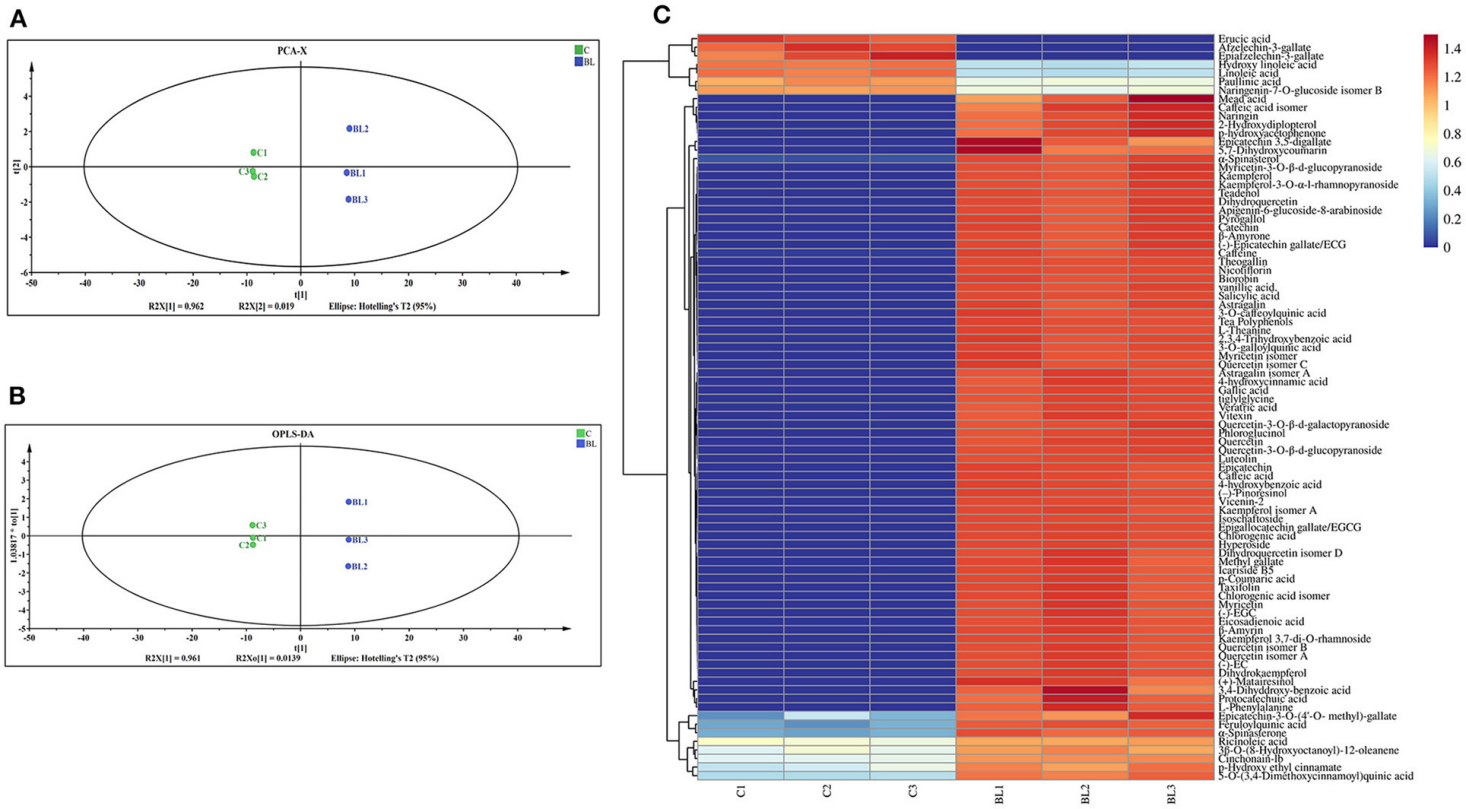
Chemical components of Tibetan tea after fermentation with *Bacillus licheniformis*. **(A)** PCA score plot; **(B)** OPLS-DA score plot; **(C)** heatmap of differential metabolic markers. PCA, principal component analysis; OPLS-DA, orthogonal partial least squares-discriminant analysis.

Changes in the chemical components of fermented and non-fermented Tibetan tea were compared using non-targeted metabolic analysis. Total ion chromatograms for both groups of samples were displayed in the Supplementary Figure S2. Ninety-eight metabolites were detected in both groups of teas. The PCA revealed distinct regions in the distribution of the principal components of Tibetan tea metabolites after intensive fermentation with *B. licheniformis* when compared with the control group (Figure 1A). This finding indicated a marked difference in the chemical composition of the fermented Tibetan tea and the control group. The model parameters were PC1: 96.2% and PC2: 1.9%. Moreover, the total variance of the samples was 98.1%, indicating a good model fit. The distribution of the control and intensively fermented Tibetan tea groups was evident in the OPLS-DA score plot (Figure 1B), indicating considerable differences in the chemical compositions. Additionally, a visual heatmap of the dynamic changes in 87 differential metabolites was created using VIP ≥ 1 as the screening criterion (Figure 1C). Among these 87 metabolites (Supplementary Table S1), flavonols (mainly kaempferol, quercetin, and myricetin), catechins, phenolic acids (including gallic acid, caffeic acid, ferulic acid, and salicylic acid), terpenoids, alkaloids, and lipids were identified. In general, the levels of fatty acids and some catechins in Tibetan tea decreased, whereas those of other metabolites increased after *B. licheniformis* enhanced fermentation.

### 3.2 *In vivo* antioxidant activity

**Figure 2:**
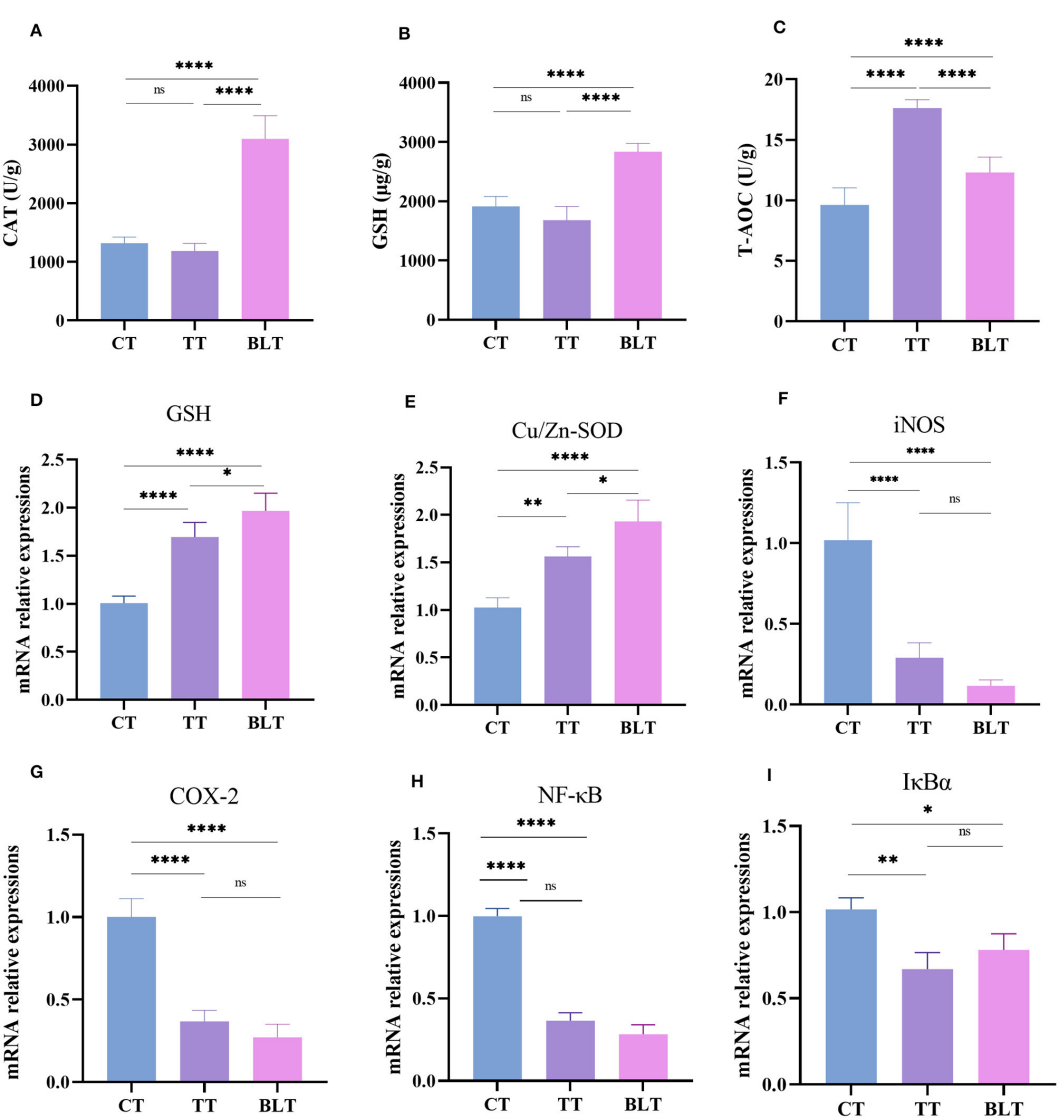
Administration of Tibetan tea by fermentation with *Bacillus licheniformis* promotes the antioxidant and anti-inflammatory capacity of the liver. **(A–C)** antioxidant enzymes; **(D–I)** expression of antioxidant and inflammatory related genes. *p < 0.05; **p < 0.005; ****p < 0.0001.

The contents of the antioxidant enzymes (CAT, GSH, and T-AOC) in the liver of mice were determined to evaluate the antioxidant activity of Tibetan tea after intensive fermentation with *B. licheniformis*. Oral gavage of Tibetan tea increased the antioxidant activity in mice (Figures 2A–C). Additionally, CAT and GSH levels were considerably increased (*p* < 0.01) in BLT compared to TT, with 3,097 U/g (*p* < 0.01) and 2,836 μg/g (*p* < 0.01), respectively. The content of T-AOC increased significantly from 9.631 U/g in C to 17.62 U/g in TT (*p* < 0.01) and 12.31 U/g in BLT (*p* < 0.01).

### 3.3 Expression levels of critical genes in mouse liver

The liver has metabolic and antioxidant functions and exhibits good antioxidant activities. Therefore, changes in the expression levels of antioxidant and inflammatory genes in the liver were measured. Glutathione (*GSH*) and Cu/Zn superoxide dismutase (*Cu/Zn-SOD*) genes in the liver of mice were upregulated (*p* < 0.01) after Tibetan tea administration, with BLT group showing upregulated expression (*p* < 0.05) (Figures 2D–I). Treatment with Tibetan tea markedly decreased the expression levels of the pro-inflammatory genes (inducible nitric oxide synthase, *iNOS*; cyclooxygenase-2, *COX-2*) in the liver of the mice. In addition, the mRNA expression levels of inhibitor kappa B-alpha (*I*κ*Bα*) and nuclear factor κB (*NF-*κ*B*) were decreased. However, there was no significant difference between BLT and TT group.

### 3.4 Effect of fermented Tibetan tea on colon histomorphology

**Figure 3:**
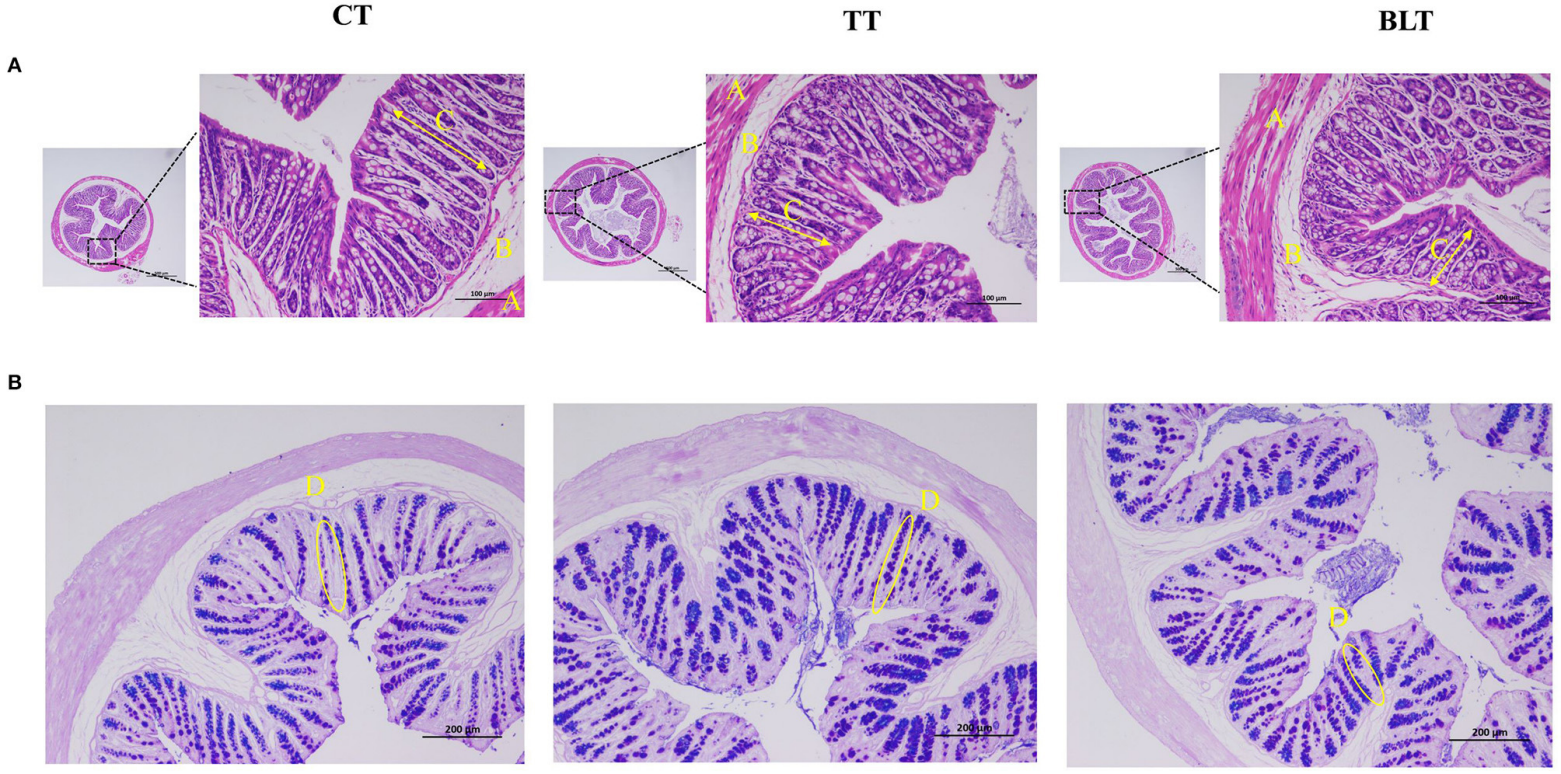
Effects of Tibetan tea after fermentation with *Bacillus licheniformis* on colon development **(A)** Hematoxylin and eosin-stained sections of colon tissue; **(B)** Alcian blue periodic acid–Schiff (AB/PAS)-stained sections of colon tissue.

Colon tissue development was evaluated using H&E and AB-PAS staining. Representative micrographs of staining results of colonic sections are presented in Figure 3. The colonic mucosal muscle layer was shown to be intact from the inside to the outside, with neatly placed glands and an unbroken crypt, and there was no evidence of inflammatory cell infiltration. The structural stratification of the colonic canal wall was evident. In addition, AB-PAS staining revealed that goblet cells in the colon were arranged in an orderly fashion and increased in number after the Tibetan tea intervention.

### 3.5 Impact of fermented Tibetan tea on expression of colonic immunity-related genes

**Figure 4:**
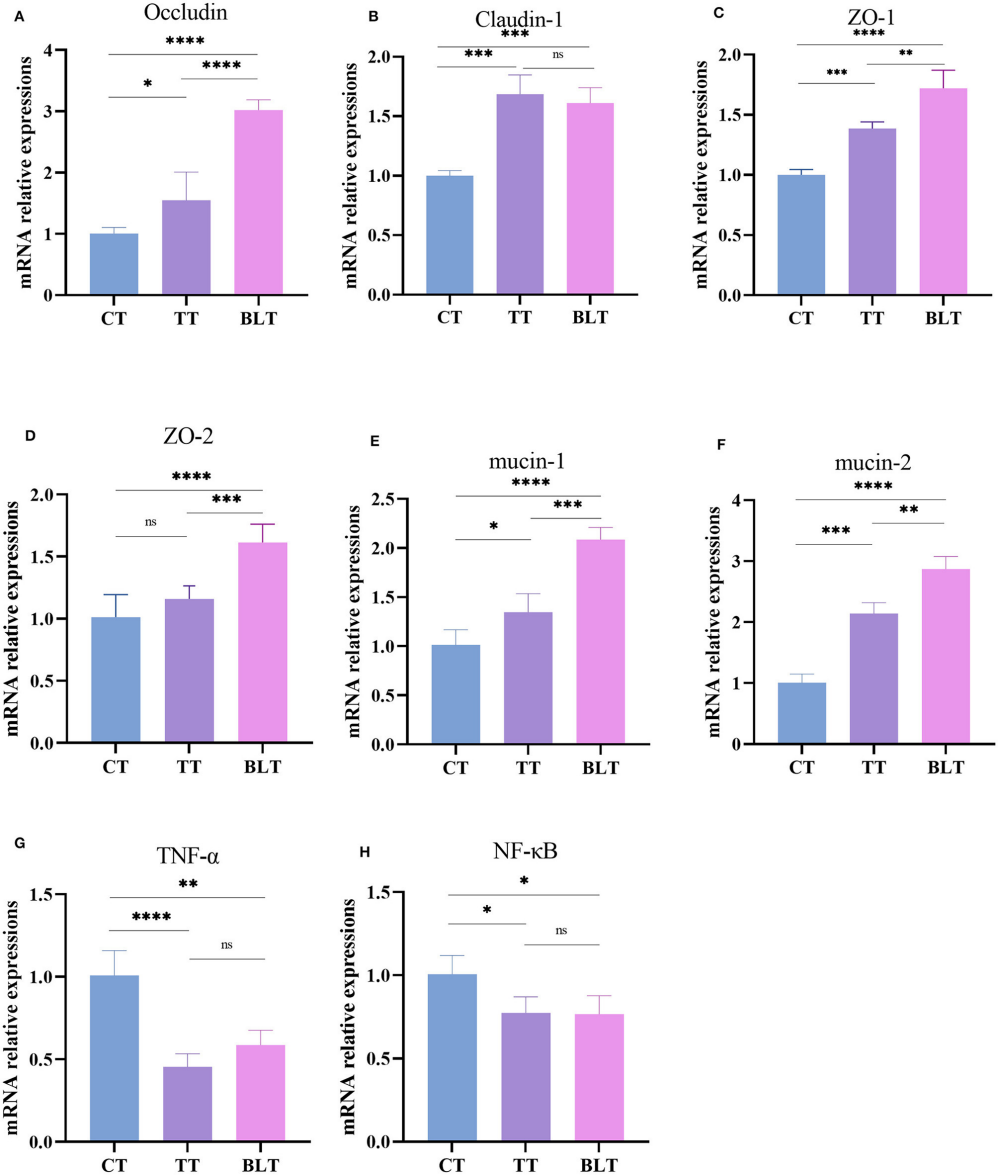
Expression level of intestinal barrier and inflammatory related genes in the colon. **(A)** *Occludin*; **(B)** *Claudin-1*; **(C)** *ZO-1*; **(D)** *ZO-2*; **(E)** *mucin-1*; **(F)** *mucin-2*; **(G)** *TNF-*α; **(H)** *NF-*κ*B*. *p < 0.05; **p < 0.005; ***p < 0.001; ****p < 0.0001.

The effects of Tibetan tea on inflammatory reactions and the intestinal barrier were investigated (Figure 4). Intensive fermentation of Tibetan tea with *B. licheniformis* substantially increased the expression of *occludin, claudin-1, ZO-1*, and *ZO-2*, which are essential genes in the tight junctions (TJs) of the intestine. Mucin plays a crucial role in the formation of the mucus barrier (Yeom et al., 2020). The levels of mucin 1 and mucin 2 increased substantially in BLT, with a 2.09- and 2.87-fold higher expression compared to CT, indicating that intensive fermentation promoted mucin expression in the colon. Additionally, Tibetan tea intervention reduced the expression of *TNF-*α and *NF-*κ*B* in the colon, indicating a reduction in inflammation. However, there was no significant difference observed between TT and BLT.

### 3.6 Effects of intensively fermented Tibetan tea on the gut microbiota

A total of 1,434,667 reads were obtained from the raw data by sequencing. Filtering Raw Tags for low quality and short length resulted in 1,414,584 reads. The final ASVs were then obtained based on the valid data by noise reduction through DADA2 and filtering out sequences. The average valid tags per sample was 66,379 reads, with an efficiency rate of 83%. The common and distinctive ASVs between various groups were assessed based on the results of ASVs acquired by noise reduction and study requirements.

**Figure 5:**
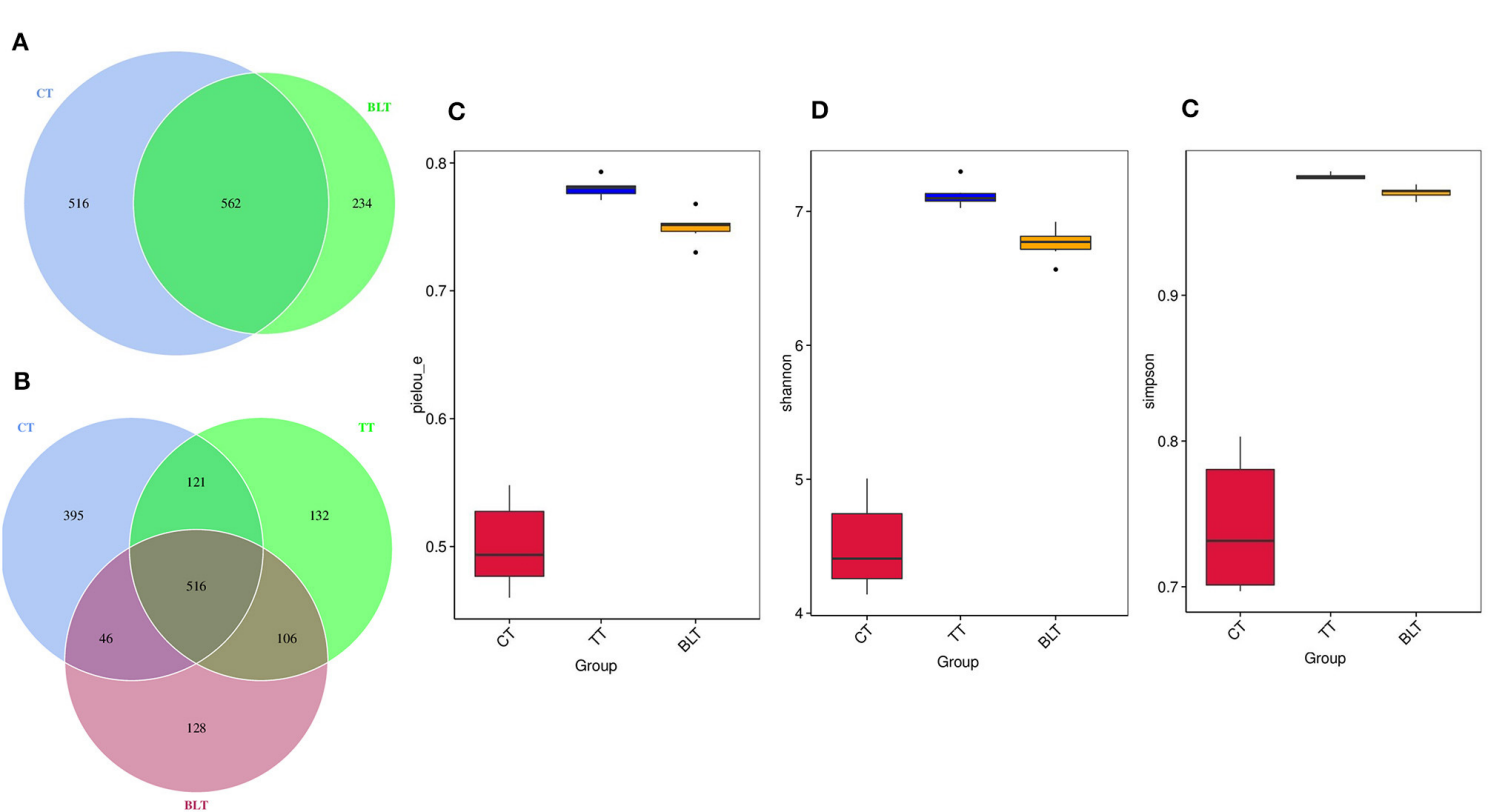
Effect of Tibetan tea on gut microbiota composition. **(A, B)** Venn plot of amplicon sequence variants (ASVs): each circle in the figure represents a group, the number of overlapping circles represents the number of ASVs shared between the groups, and the number of unique ASVs without overlapping parts represents the number of ASVs unique to the group; **(C)** Pielou_e index; **(D)** Shannon index; **(E)** Simpson index.

As shown in Figures 5A, B, 1,444 ASVs were detected in the whole sample, 1,078, 875, and 796 in CT, TT, and BLT, respectively, and 395, 132, and 128 unique ASVs. There were fewer ASVs in BLT than in CT, indicating that the intensive fermentation of Tibetan tea by *B. licheniformis* lowered the quantity of ASVs in the intestinal flora.

Alpha diversity contains several indicators that reflect the richness and diversity of microbial communities. The Pielou_e index represents microbial evenness, with a larger Pielou_e representing more even species. The Pielou_e index of Tibetan tea was higher (*p* < 0.01) (Figure 5C). The Shannon and Simpson indices reflect microbial diversity, with the Simpson index being more sensitive to species evenness and the Shannon index being more sensitive to species richness. After receiving Tibetan tea treatment, the Shannon and Simpson indices both showed an upward trend (Figures 5D, E), indicating that species richness and homogeneity of intestinal flora improved.

**Figure 6:**
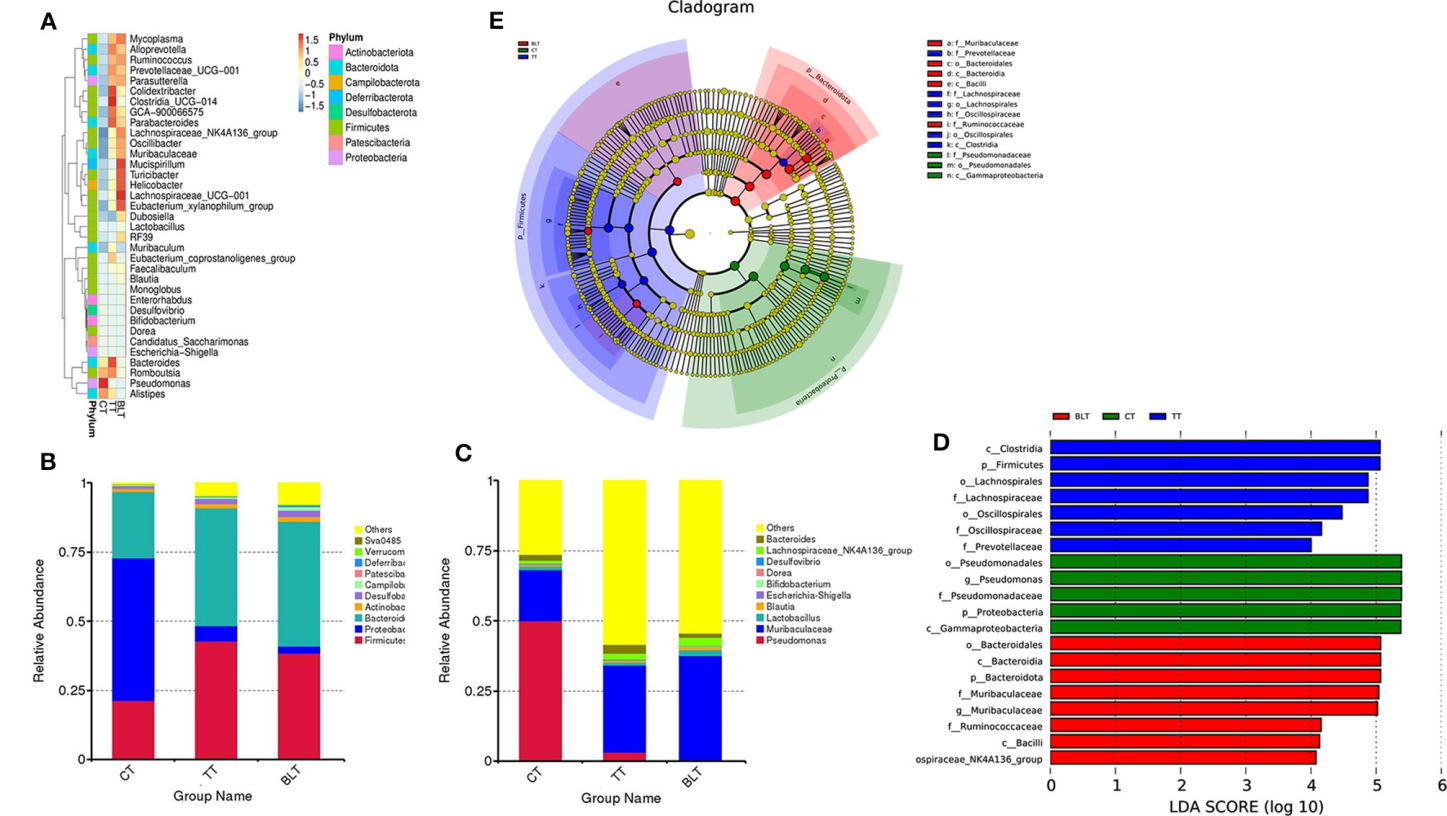
Changes in the relative abundance of bacteria in the microbial community after ingesting Tibetan tea. **(A)** Heatmap of top 35 genera in genus level abundance; **(B)** Bacterial taxonomic profiling at the phylum and genus **(C)** level; linear discriminant analysis (LDA) effect size (LEfSe) diagram of the significant taxonomies between three groups, including LDA value distribution histograms **(D)** and cladistics **(E)**.

Based on the species annotation and abundance information of all samples at the genus level, the top 35 genera in terms of abundance were selected and clustered at both species and sample levels based on their abundance information in each sample and plotted as a heat map into eight phyla (Figure 6A). At the phylum level (Figure 6B), *Proteobacteria* made up 51.5% of the total bacteria in the CT group, with higher percentages of *Firmicutes* and *Bacteroidota* (21.3 and 24.2%, respectively), whereas the sum of the relative abundance of *Firmicutes* and *Bacteroidota* in the TT and BLT groups accounted for more than 80% of the total bacteria and was the absolute dominant phylum in both groups. In contrast, after intensive fermentation, the relative abundance of the *Proteobacteria* reduced dramatically (*p* < 0.01) and it was no longer the dominant bacterium in the intestinal flora at the phylum level.

At the genus level, the relative abundance of *Pseudomonas* in the CT group reached 50% (Figure 6C). However, it was only 3.2 and 0.4% in the TT and BLT groups, respectively, indicating that intensively-fermented Tibetan tea substantially reduced the relative abundance of *Pseudomonas* in the intestinal flora. The relative abundance of *Muribaculaceae* in the CT group was only 18.2%, whereas that in the TT and BLT groups was 31.1 and 37.2%, respectively, showing a marked upward trend compared to the CT group. The dominant genera in the BLT group were *Muribaculaceae* (37.2%), *Lachnospiraceae_NK4A136_group* (3.0%), *Lactobacillus* (2.2%), and *Dubosiella* (2.1%). The relative abundance of these beneficial bacteria was substantially higher in the BLT group than in the CT and TT groups.

The three groups were evaluated using LEfSe to identify biomarkers with statistical differences between the groups. The length of the bar chart displays the effect size of the various species (i.e., the LDA score). LEfSe analysis revealed 20 biomarkers differed among the three groups (Figures 6D, E). There were 13 characteristic microbes in the Tibetan tea group, of which *f_Prevotellaceae, f_Oscillospiraceae, o_Oscillospirales, f_Lachnospiraceae, o_Lachnospirales, p_Firmicutes, c_Clostridia* were shown to be abundant in the TT group. Moreover, some bacteria with statistically significant differences, including the *g_Lachnospiraceae_NK4A136_group, c_Bacilli, f_Ruminococcaceae, g_Muribaculaceae, f_Muribaculaceae, p_Bacteroidota, c_Bacteroidia, o_Bacteroidales*, were observed in the intestinal flora of the BLT group and became biomarkers in the BLT group.

**Figure 7:**
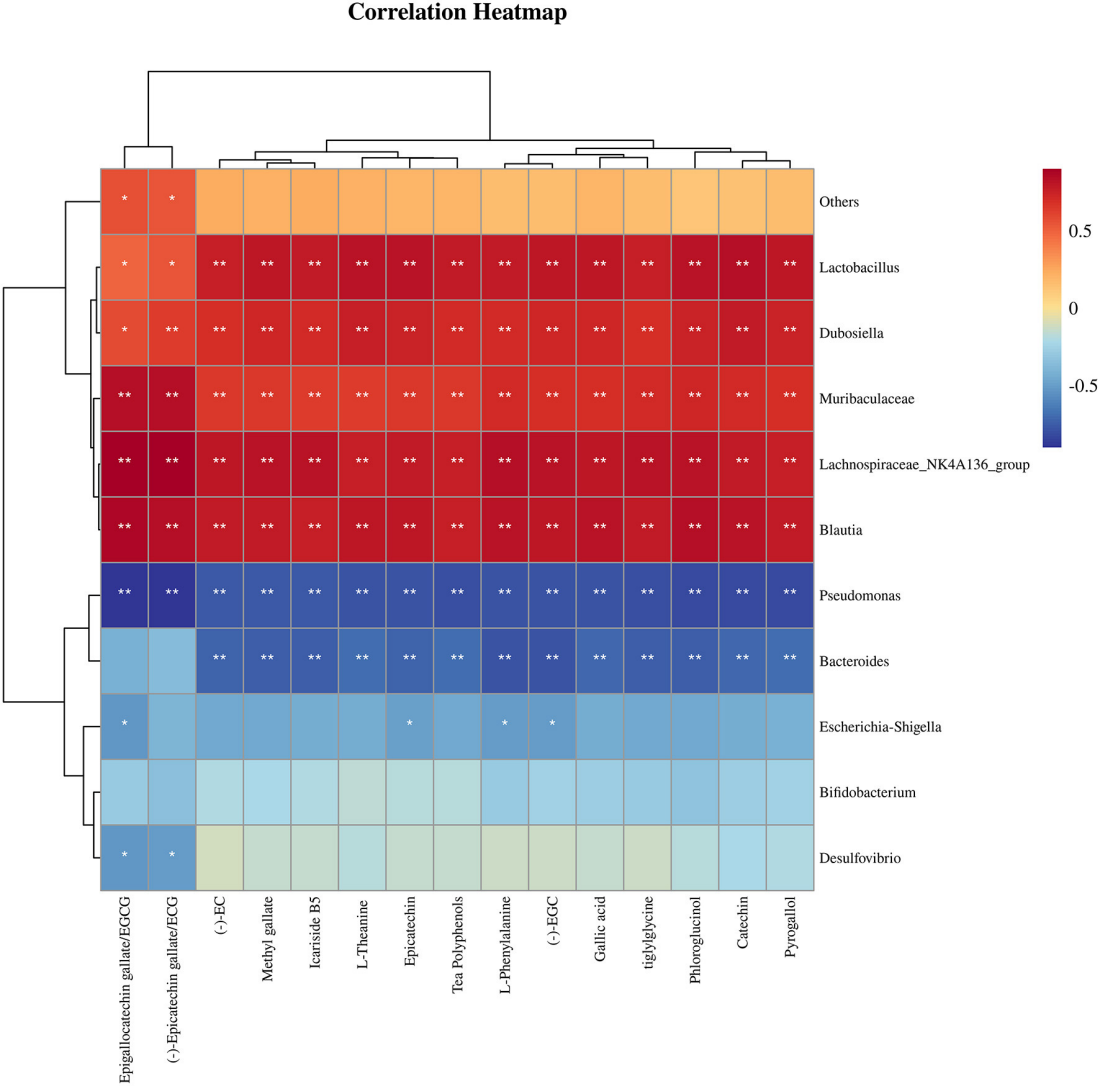
Correlation analysis between chemical substances with substantial changes in relative content and the most differentially-abundant taxa of mouse intestinal microflora after ingestion of Tibetan tea intensively-fermented using *Bacillus licheniformis*. *p < 0.05; **p < 0.005.

Figure 7 illustrates the correlation between the 15 compounds with substantial increases in their relative concentration in fortified, fermented Tibetan tea and the 10 genera with the most significant changes in the relative abundance of the intestinal microbes. *Lactobacillus, Dubosiella, Muribaculaceae, Lachnospiraceae_NK4A136_group*, and *Blautia* were positively correlated with chemical substance content (*p* < 0.01); *Pseudomonas* was negatively correlated with chemical substance content (*p* < 0.01); *Escherichia–Shigella* was correlated with EGCG, Epicatechin, L–Phenylalanine; (–) –EGC content was negatively correlated (*p* < 0.05); and *Desulfovibrio* was negatively correlated with EGCG and ECG content (*p* < 0.05).

## 4 Discussion

Intensified fermentation increases the chemical composition in Tibetan tea. The biological activity of tea is directly correlated to its chemical composition. The functional components of Tibetan tea are primarily derived from three sources. First, they are formed during the natural growth of tea plants. Second, when produced, Tibetan tea interacts with microorganisms in high-temperature and high-humidity settings through pile-fermentation, drying, and other processes. The tea and its components undergo a complex series of transformations, developing a range of new compounds or metabolites. Third, the metabolic compounds are produced by the tea's microbial community (Li et al., 2018; Zheng et al., 2020; Liu et al., 2022). This study used *B. licheniformis* for the enhanced fermentation of Tibetan tea. The chemical composition results showed that after fermentation, the main metabolites in Tibetan tea included polyphenols, alkaloids, terpenoids, amino acids, and lipids.

The antioxidant capacity of tea is primarily based on the biological activity of polyphenols. After intensive fermentation, the content of polyphenols in Tibetan tea became richer and more diverse, with up to 55 types, including flavonoids, catechins, and phenolic acids. Naringin, a flavanone glycoside with anti-inflammatory and hepatoprotective properties, has been detected in fermented Tibetan tea (Shilpa et al., 2023). Although catechins are the main contributors to the antioxidant effects of tea, they also have various biological activities, such as hypolipidemic and immune regulation properties (Tang et al., 2019). After fermentation, Tibetan tea contains various phenolic acids, including gallic, caffeic, p-coumaric, chlorogenic, and salicylic acids. Amino acids in Tibetan tea play an important role in the sensory quality of tea infusions.

As hydrophobic metabolites, lipids affect the quality of tea infusions. Untargeted metabolomics analysis based on UHPLC-Q-TOF showed that the levels of paullinic, erucic, and linoleic acids decreased after fermentation, which may be due to their decomposition and conversion into aromatic substances during fermentation. However, the levels of eicosadienoic acid, ricinoleic acid, mead acid, and 3β-O- (8-hydroxyoctanoyl)-12-oleanene increased after fermentation.

Tea is a major source of alkaloids and the presence of alkaloids contributes considerably to its sensory quality. Of the alkaloids, caffeine has a bitter flavor and works together with catechins and amino acids to make the tea infusion bitter and then mellow (Bi et al., 2016). In addition, 5,7-dihydroxycoumarin and α-spinasterol have antibacterial activities, and α-spinasterol can inhibit the activities of *COX-1* and *COX-2* (Yang et al., 2017). Four terpenoids, including β-amyrone, icariside B5, β-amyrin, and 2-hydroxydiplopterol, which are important for tea aroma, were detected in fermented Tibetan tea. β-Amyrone is a triterpene that exerts anti-inflammatory activities by inhibiting the expression of *COX-2* (De Almeida et al., 2015). After intensive fermentation, these chemical components in Tibetan tea become the characteristic components, enhancing the anti-oxidation, anti-inflammation, and other health benefits of the tea. To confirm the enhancement of the health functions of Tibetan tea by intensive fermentation with *B. licheniformis*, relevant indices were examined in this study.

After fermentation, the antioxidant capacity of Tibetan tea was increased. After food is consumed, the liver participates in synthesis and metabolism (Kandimalla et al., 2016). Antioxidant enzymes, such as CAT and GSH, which maintain normal oxidation levels in the body, are part of the endogenous antioxidant system in the liver. Decreased levels of these antioxidant-active substances can negatively affect the liver (Wu et al., 2021b). The intragastric administration of Tibetan tea increased the levels of the antioxidant enzymes, CAT and GSH, in the livers of mice, including an increase in T-AOC levels. Specifically, intensive fermentation with *B. licheniformis* had a more marked effect on the increase in CAT and GSH levels than the non-fermented Tibetan tea group. This result suggests that the ingestion of Tibetan tea fermented using microorganisms enhances the antioxidant capacity of the liver by boosting antioxidant enzyme activity. In this study, Tibetan tea considerably increased the mRNA expression of *GSH* and *Cu/Zn-SOD* and the effect was greater with fermented Tibetan tea. This observation indicates that the intensive fermentation of Tibetan tea with a probiotic led to an improvement in its antioxidant capacity.

*NF-*κ*B* is a transcription factor that can regulate several genes involved in inflammation and secretes pro-inflammatory cytokines, which cause inflammation. Inflammatory factors, *iNOS* and *COX-2*, participate in the biosynthesis of NO and prostaglandins, respectively, and are downstream of the NF-κB pathway. When liver damage occurs, the NF-κB pathway is activated, resulting in high expression of the downstream inflammatory factors, *iNOS* and *COX-2*, thereby promoting liver injury and escalating liver inflammation. The activation of NF-κB, a major signaling pathway regulating the transcription of inflammatory mediators in macrophages, leads to the expression of the *I*κ*B*α gene (Oh et al., 2020; Wu et al., 2022). Compared to the control group, the administration of Tibetan tea resulted in a substantial decrease in the expression of *I*κ*B*α, *iNOS, COX-2*, and *NF-*κ*B* genes, indicating that Tibetan tea inhibited the NF-κB pathway and suppressed inflammation. Intense fermentation of Tibetan tea using *B. licheniformis* substantially increased the levels of antioxidant enzymes and upregulated the expression levels of antioxidant-related genes, demonstrating that probiotic fermentation enhanced the antioxidant properties of the tea. There was no discernible difference between the Tibetan tea and enhanced fermentation group regarding the inhibitory effects on pro-inflammatory genes.

The mucosal layer, epithelial cells, and tight intercellular junctions form the intestinal barrier, which shields the body from potentially harmful metabolites, bacteria, and antigens. Immune regulation of the intestinal barrier is crucial. More precisely, the physical and biological functions of the intestinal epithelium, such as the expression of mucus and epithelial and endothelial connections, are crucial for maintaining the intestinal barrier (Tilg et al., 2022). The colon tissue has a more abundant microbiota, with a stronger ability to ferment to produce various microbial metabolites. This enriched microbiota and microbial fermentation products are important for gut health and host metabolism (Zhang et al., 2019). Therefore, the changes in colon-related parameters were measured. Staining of colonic sections revealed that Tibetan tea treatment increased the number of goblet cells, indicating that the intestinal barrier was effectively protected. These specialized cells can secrete mucus, thereby facilitating lubrication and safeguarding the intestinal mucosa. After Tibetan tea treatment, the mRNA expression of the intestinal shielding-related genes, *occludin, claudin-1, mucin-1, mucin-2, ZO-1*, and *ZO-2*, in the colon increased substantially. The upregulation was more pronounced in the BLT group. This finding demonstrates that Tibetan tea fermented using *B. licheniformis* enhances TJs and intestinal mucosal barriers by upregulating TJ protein and mucin-related mRNA expression.

*TNF-*α, a pro-inflammatory factor that can be expressed through the NF-κB pathway, triggers inflammation. Increased levels of *TNF-*α also induce mucosal inflammation and mechanical barrier damage in the intestine (Chen et al., 2021). In the current study, the expression of *TNF-*α and *NF-*κ*B* genes in the colon was inhibited by the administration of Tibetan tea, demonstrating its anti-inflammatory effect. Tibetan tea and intensively fermented Tibetan tea showed no discernible differences in inhibition.

Enhanced fermented Tibetan tea promotes a healthy function by regulating intestinal flora. There are extensive and intimate interactions between the liver and gut, and the disruption of the intestinal flora leads to the overgrowth of harmful bacteria, which causes immune abnormalities of the intestinal barrier. The intestinal microbiota in humans has emerged as a key player in the regulation of the intestinal barrier and metabolic diseases. Intestinal homeostasis can be directly influenced by dietary composition and nutrient intake, which can modify the intestinal structure. Components that are difficult for the human body to absorb directly operate in the presence of the intestinal flora (Forgie et al., 2019; Lin et al., 2019). Therefore, the effect of fermented Tibetan tea on the intestinal microflora was studied further. The species richness and evenness of the intestinal microflora in mice were increased substantially by administering Tibetan tea, indicating that Tibetan tea had a significant intervention effect. Furthermore, β-diversity analysis revealed that intervention with fortified fermented Tibetan tea substantially increased the proportion of beneficial microorganisms, such as *Muribaculaceae, Lachnospiraceae_NK4A136_group, Lactobacillus*, and *Dubosiella*, in the intestinal tract. Moreover, it decreased the proportion of pathogenic bacteria, such as *Proteobacteria*, which has a noteworthy pro-inflammatory effect on the mucosal surface and was observed to have a disruptive effect on the epithelial barrier in animal models (Tilg et al., 2022). Host and microbial genomes jointly regulate and produce metabolites, such as SCFAs and bile acids, to maintain the health of living organisms. The related genera of SCFA-producing bacteria in the intestine and their metabolically-produced SCFAs play critical roles in the maintenance of host intestinal barrier functions (Sina et al., 2009; Li et al., 2021). *Muribaculaceae*, which are beneficial microorganisms in the gut flora of mice and members of the *Bacteroidetes*, extend the lifespan of mice and produce acetate and propionic acid by fermentation. Increased *Muribaculaceae* abundance prevents lipid metabolism disorders, inflammation, and intestinal barrier malfunction (Wu et al., 2021a; Li W. et al., 2023). *Lachnospiraceae_NK4A136_group* is a butyrate-producing bacterium that ferments plant polysaccharides into SCFAs and ethanol. It is a potential probiotic with anti-inflammatory properties. It also contributes to the repair of the intestinal mucosa and is associated with bile acid metabolism. Butyrate-producing gut bacteria can improve host immunity, guarantee barrier integrity, and control energy metabolism. Butyrate can influence neutrophil function and metastasis, inhibit inflammatory cytokines in vascular cells, increase the expression of TJ proteins in colonic epithelial cells, and decrease the expression of cytokines and chemokines in human immune cells (Ma et al., 2020; Zhong et al., 2021). As a probiotic, *Lactobacillus* can improve the intestinal barrier in several ways, such as by boosting mucus formation, stimulating cathelicidin and antibacterial factor release, preventing microbial elements involved in epithelial adherence (for example, in secretory immunoglobulin A), and increasing the expression of closure and TJ proteins.

**Figure 8:**
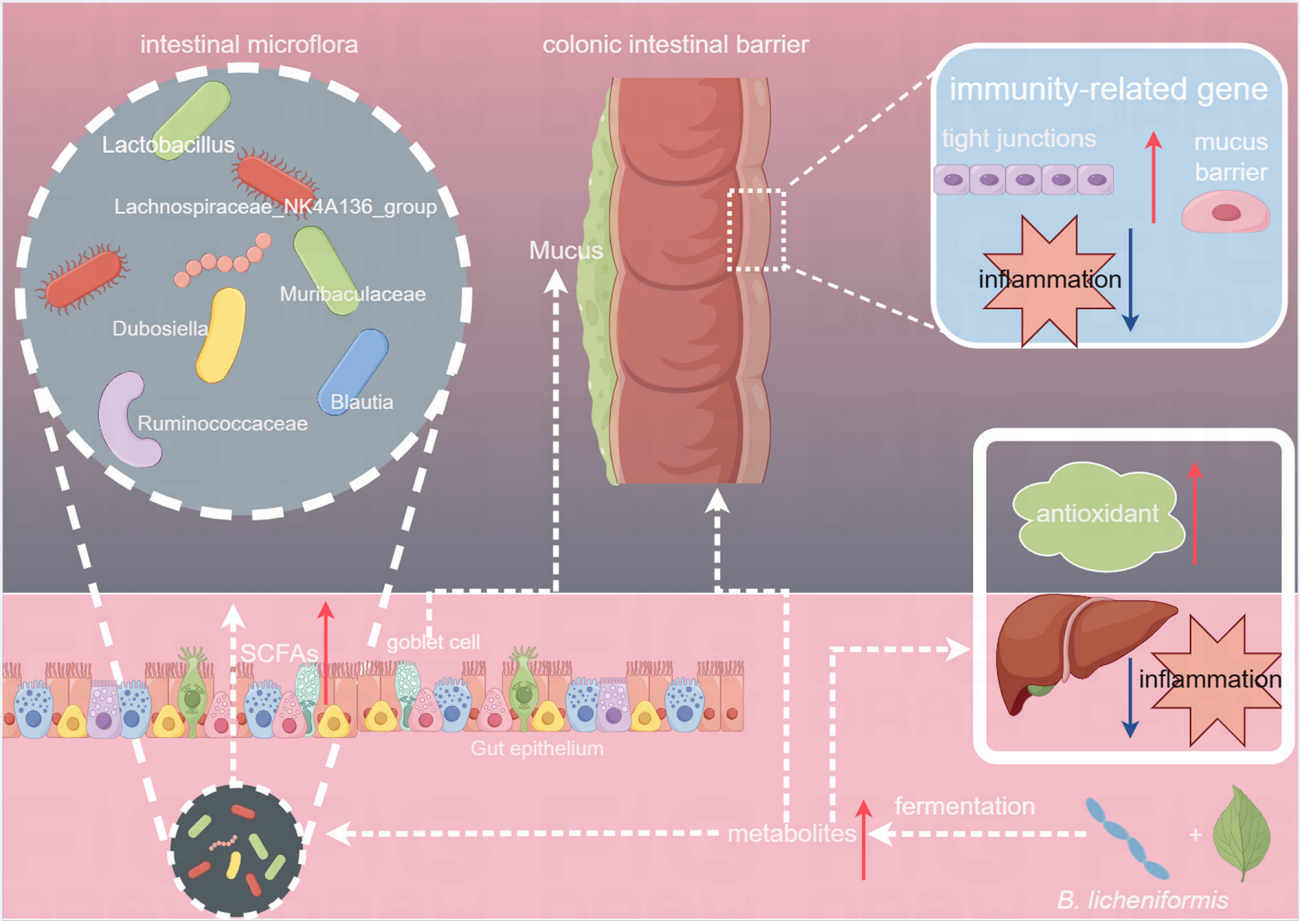
Effects of Tibetan tea regulation after fermentation with *Bacillus licheniformis* in the intestine and liver.

Previous studies have revealed that *Dubosiella* can produce SCFAs, which are crucial in regulating metabolism, enhancing intestinal immunity, and promoting anti-inflammatory responses in the body (Ai et al., 2021). In the BLT group, LEfSe analysis revealed that *Lachnospiraceae_NK4A136_group, Bacilli, Ruminococcaceae*, and *Bacteroidota* were critical biomarkers. *Ruminococcaceae* are SCFA-producing bacteria that produce formic and acetic acids. The abundance of *Ruminococcaceae* is positively correlated with intestinal motility and a high abundance reduces intestinal cell damage and proinflammatory cytokine production in the colon (Li L. et al., 2023). Therefore, the ingestion of Tibetan tea, which underwent enhanced fermentation using *B. licheniformis*, increased the relative abundance of SCFA-producing genera in the mouse colon, promoting intestinal development, preserving the integrity of the intestinal mucosal barrier, and safeguarding intestinal morphology (Figure 8).

## 5 Conclusions

The probiotic *B. licheniformis* was used to intensify the fermentation of Tibetan tea. The results indicated that intensive fermentation of Tibetan tea led to an increase in bioactive substances such as polyphenols, alkaloids, terpenoids, and amino acids while reducing catechins and lipids. The fermentation process significantly boosted the antioxidant enzymes (CAT, GSH, T-AOC) in the liver and up-regulated the expression of antioxidant-related genes, such as *GSH* and *Cu/Zn SOD*, indicating enhanced antioxidant capacity. Consumption of intensified fermented Tibetan tea also positively influenced intestinal flora by increasing the abundance of probiotic bacteria such as *Lachnospiraceae_NK4A136_group, Muribaculaceae*, and *Lactobacillus*, while decreasing harmful bacteria like *Proteobacteria* and *Pseudomonas*. Furthermore, fermentation of Tibetan tea with *B. licheniformis* led to an increase in SCFAs-producing genera in the gut microbiota, reinforcing the integrity of the intestinal barrier, enhancing mucosal immunity, and ultimately improving intestinal immunomodulation. These effects were supported by the detection of elevated expression levels of genes related to the intestinal barrier in the colon. Therefore, these insights provided a compelling empirical framework for advancing the production methodologies of Tibetan tea to maximize its health benefits.

Previous research in the field of dark tea has predominantly focused on Pu-erh tea, while studies on Tibetan tea have primarily centered on its bioactive properties. Research on microbial fermented tea has mainly delved into fungi and molds, with limited attention on bacteria. In this study, *Bacillus licheniformis*, a bacterium isolated from Tibetan tea, was used to ferment Tibetan tea, examining the impact of this process on its chemical composition and health benefits. An intriguing discovery in this study of the impact of *B. licheniformis* on the fermentation process was the significant increase in SCFAs-producing genera in the gut, potentially enhancing the intestinal barrier. However, one downside regarding our methodology was the lack of examination of SCFA content in the intestine, which warrants further investigation in future studies.

## Data availability statement

The datasets presented in this study can be found in online repositories. The names of the repository/repositories and accession number (s) can be found in the article/Supplementary material.

## Ethics statement

The animal study was approved by the Laboratory Animal Ethics Committee (approval number: swmu20220137). The study was conducted in accordance with the local legislation and institutional requirements.

## Author contributions

HZ: Conceptualization, Methodology, Project administration, Supervision, Writing – review & editing, Data curation, Software, Visualization. XZ: Data curation, Methodology, Software, Visualization, Writing – original draft. CSh: Writing – original draft, Investigation. ZA: Investigation, Writing – original draft. XC: Investigation, Writing – original draft. CSo: Investigation, Writing – original draft. MM: Project administration, Writing – review & editing. TW: Writing – review & editing, Formal analysis. JM: Resources, Writing – original draft. MH: Resources, Writing – review & editing. YM: Formal analysis, Software, Supervision, Writing – review & editing. NW: Supervision, Conceptualization, Methodology, Project administration, Writing – review & editing.

## References

[B1] Adak A. Khan M. R. (2019). An insight into gut microbiota and its functionalities. Cell. Mol. Life Sci. 76, 473–493. 10.1007/s00018-018-2943-4 30317530 PMC11105460

[B2] Ai X. Wu C. Yin T. Zhur O. Liu C. Yan X.. (2021). Antidiabetic function of *Lactobacillus fermentum* MF423-fermented rice bran and its effect on gut microbiota structure in type 2 diabetic mice. Front. Microbiol. 12:682290. 10.3389/fmicb.2021.682290 34248898 PMC8266379

[B3] Bi W. He C. Ma Y. Shen J. Zhang L. H. Peng Y.. (2016). Investigation of free amino acid, total phenolics, antioxidant activity and purine alkaloids to assess the health properties of non-Camellia tea. Acta Pharm. Sin. B 6, 170–181. 10.1016/j.apsb.2015.11.003 27006902 PMC4788713

[B4] Bond T. Derbyshire E. (2019). Tea compounds and the gut microbiome: findings from trials and mechanistic studies. Nutrients 11:2364. 10.3390/nu11102364 31623411 PMC6835862

[B5] Chen G. Xie M. Wan P. Chen D. Ye H. Chen L.. (2018). Digestion under saliva, simulated gastric and small intestinal conditions and fermentation in vitro by human intestinal microbiota of polysaccharides from Fuzhuan brick tea. Food Chem. 244, 331–339. 10.1016/j.foodchem.2017.10.074 29120790

[B6] Chen Y. Yang B. Stanton C. Ross R. P. Zhao J. Zhang H.. (2021). *Bifidobacterium pseudocatenulatum* ameliorates DSS-induced colitis by maintaining intestinal mechanical barrier, blocking proinflammatory cytokines, inhibiting TLR4/NF-κB signaling, and altering gut microbiota. J. Agric. Food Chem. 69, 1496–1512. 10.1021/acs.jafc.0c06329 33512996

[B7] De Almeida P. D. Boleti A. P. Rüdiger A. L. Lourenço G. A. Da Veiga Junior V. F. Lima E. S. (2015). Anti-inflammatory activity of triterpenes isolated from *Protium paniculatum* oil-resins. Evid. Based Complement. Alternat. Med. 2015:293768. 10.1155/2015/293768 27034686 PMC4806667

[B8] Forgie A. J. Fouhse J. M. Willing B. P. (2019). Diet-microbe-host interactions that affect gut mucosal integrity and infection resistance. Front. Immunol. 10:1802. 10.3389/fimmu.2019.01802 31447837 PMC6691341

[B9] Kandimalla R. Kalita S. Saikia B. Choudhury B. Singh Y. P. Kalita K.. (2016). Antioxidant and hepatoprotective potentiality of *Randia dumetorum* Lam. leaf and bark via inhibition of oxidative stress and inflammatory cytokines. Front. Pharmacol. 7:205. 10.3389/fphar.2016.00205 27471465 PMC4943931

[B10] Li L. Qiu N. Meng Y. Wang C. Mine Y. Keast R.. (2023). Preserved egg white alleviates DSS-induced colitis in mice through the reduction of oxidative stress, modulation of infl ammatory cytokines, NF-κB, MAPK and gut microbiota composition. Food Sci. Human Wellness 12, 312–323. 10.1016/j.fshw.2022.07.021

[B11] Li N. Dai Z. Wang Z. Deng Z. Zhang J. Pu J.. (2021). Gut microbiota dysbiosis contributes to the development of chronic obstructive pulmonary disease. Respir. Res. 22:274. 10.1186/s12931-021-01872-z 34696775 PMC8543848

[B12] Li Q. Chai S. Li Y. Huang J. Luo Y. Xiao L.. (2018). Biochemical components associated with microbial community shift during the pile-fermentation of Primary Dark Tea. Front. Microbiol. 9:1509. 10.3389/fmicb.2018.01509 30042750 PMC6048958

[B13] Li W. Wang Z. Cao J. Dong Y. Chen Y. (2023). Melatonin improves the homeostasis of mice gut microbiota rhythm caused by sleep restriction. Microbes Infect. 25:105121. 10.1016/j.micinf.2023.105121 36804006

[B14] Lin Y. Zheng X. Chen J. Luo D. Xie J. Su Z.. (2019). Protective effect of *Bruguiera gymnorrhiza* (L.) Lam. fruit on dextran sulfate sodium-induced ulcerative colitis in mice: role of Keap1/Nrf2 pathway and gut microbiota. Front. Pharmacol. 10:1602. 10.3389/fphar.2019.01602 32116661 PMC7008401

[B15] Liu Y. Huang W. Zhang C. Li C. Fang Z. Zeng Z.. (2022). Targeted and untargeted metabolomic analyses and biological activity of Tibetan tea. Food Chem. 384:132517. 10.1016/j.foodchem.2022.132517 35228002

[B16] Liu Y. Wang X. Chen Q. Luo L. Ma M. Xiao B.. (2020). *Camellia sinensis* and *Litsea coreana* ameliorate intestinal inflammation and modulate gut microbiota in dextran sulfate sodium-induced colitis mice. Mol. Nutr. Food Res. 64:e1900943. 10.1002/mnfr.201900943 31951100

[B17] Liu Z. Bruins M. E. Ni L. Vincken J. P. (2018). Green and black tea phenolics: bioavailability, transformation by colonic microbiota, and modulation of colonic microbiota. J. Agric. Food Chem. 66, 8469–8477. 10.1021/acs.jafc.8b02233 30020786

[B18] Ma L. Ni Y. Wang Z. Tu W. Ni L. Zhuge F.. (2020). Spermidine improves gut barrier integrity and gut microbiota function in diet-induced obese mice. Gut Microbes 12, 1–19. 10.1080/19490976.2020.1832857 33151120 PMC7668533

[B19] Oh W. S. Jung J. C. Choi Y. M. Mun J. Y. Ku S. K. Song C. H. (2020). Protective effects of fermented rice extract on ulcerative colitis induced by dextran sodium sulfate in mice. Food Sci. Nutr. 8, 1718–1728. 10.1002/fsn3.1460 32180979 PMC7063356

[B20] Park M. H. Yeom Y. J. Ganbat D. Kim M. K. Kim S. B. Lee Y. J.. (2023). Fermentation of *Abelmoschus manihot* extract with halophilic *Bacillus licheniformis* CP6 results in enhanced anti-inflammatory activities. Nutrients 15:309. 10.3390/nu15020309 36678181 PMC9864326

[B21] Qi N. Zhan X. Milmine J. Sahar M. Chang K. H. Li J. (2023). Isolation and characterization of a novel hydrolase-producing probiotic *Bacillus licheniformis* and its application in the fermentation of soybean meal. Front. Nutr. 10:1123422. 10.3389/fnut.2023.1123422 36969826 PMC10030947

[B22] Rowland I. Gibson G. Heinken A. Scott K. Swann J. Thiele I.. (2018). Gut microbiota functions: metabolism of nutrients and other food components. Eur. J. Nutr. 57, 1–24. 10.1007/s00394-017-1445-8 28393285 PMC5847071

[B23] Samynathan R. Thiruvengadam M. Nile S. H. Shariati M. A. Rebezov M. Mishra R. K.. (2023). Recent insights on tea metabolites, their biosynthesis and chemo-preventing effects: a review. Crit. Rev. Food Sci. Nutr. 63, 3130–3149. 10.1080/10408398.2021.1984871 34606382

[B24] Shilpa V. S. Shams R. Dash K. K. Pandey V. K. Dar A. H. Ayaz Mukarram S.. (2023). Phytochemical properties, extraction, and pharmacological benefits of naringin: a review. Molecules 28:5623. 10.3390/molecules28155623 37570594 PMC10419872

[B25] Sina C. Gavrilova O. Förster M. Till A. Derer S. Hildebrand F.. (2009). G protein-coupled receptor 43 is essential for neutrophil recruitment during intestinal inflammation. J. Immunol. 183, 7514–7522. 10.4049/jimmunol.0900063 19917676

[B26] Tan Y. Li M. Kong K. Xie Y. Zeng Z. Fang Z.. (2023). *In vitro* simulated digestion of and microbial characteristics in colonic fermentation of polysaccharides from four varieties of Tibetan tea. Food Res. Int. 163:112255. 10.1016/j.foodres.2022.112255 36596166

[B27] Tang G. Y. Meng X. Gan R. Y. Zhao C. N. Liu Q. Feng Y. B.. (2019). Health functions and related molecular mechanisms of tea components: an update review. Int. J. Mol. Sci. 20:6196. 10.3390/ijms20246196 31817990 PMC6941079

[B28] Tilg H. Adolph T. E. Trauner M. (2022). Gut-liver axis: pathophysiological concepts and clinical implications. Cell Metab. 34, 1700–1718. 10.1016/j.cmet.2022.09.017 36208625

[B29] Wang N. Mo S. Wu T. Mehmood M. A. Sun H. Tang Y.. (2023). Metabolomic analysis of fermented tibetan tea using *Bacillus circulans* and their biological activity on mice via the intestine-hepatic axis. Probiotics Antimicrob. Proteins. 15, 1653–1664. 10.1007/s12602-023-10049-7 36806153

[B30] Wu Z. Huang S. Li T. Li N. Han D. Zhang B.. (2021a). Gut microbiota from green tea polyphenol-dosed mice improves intestinal epithelial homeostasis and ameliorates experimental colitis. Microbiome 9:184. 10.1186/s40168-021-01115-9 34493333 PMC8424887

[B31] Wu Z. Ma Q. Cai S. Sun Y. Zhang Y. Yi J. (2021b). Rhus chinensis Mill. Fruits ameliorate hepatic glycolipid metabolism disorder in rats induced by high fat/high sugar diet. Nutrients 13:4480. 10.3390/nu13124480 34960032 PMC8708379

[B32] Wu Z. Sun L. Chen R. Wen S. Li Q. Lai X.. (2022). Chinese tea alleviates CCl(4)-induced liver injury through the NF-κBorNrf2 signaling pathway in C57BL-6J mice. Nutrients 14:972. 10.3390/nu14050972 35267945 PMC8912361

[B33] Yang X. Zhou J. Wang T. Zhao L. Ye G. Shi F.. (2017). A novel method for synthesis of α-spinasterol and its antibacterial activities in combination with ceftiofur. Fitoterapia 119, 12–19. 10.1016/j.fitote.2017.03.011 28351722

[B34] Yeom J. Ma S. Lim Y. H. (2020). Oxyresveratrol induces autophagy via the ER stress signaling pathway, and oxyresveratrol-induced autophagy stimulates MUC2 synthesis in human goblet cells. Antioxidants 9:214. 10.3390/antiox9030214 32150901 PMC7139292

[B35] Yong C. C. Yoon Y. Yoo H. S. Oh S. (2019). Effect of *Lactobacillus* fermentation on the anti-inflammatory potential of turmeric. J. Microbiol. Biotechnol. 29, 1561–1569. 10.4014/jmb.1906.06032 31434176

[B36] Zhang C. Liu Y. Chen S. Qiao Y. Zheng Y. Xu M.. (2019). Effects of intranasal pseudorabies virus AH02LA infection on microbial community and immune status in the ileum and colon of piglets. Viruses 11:518. 10.3390/v11060518 31195631 PMC6631256

[B37] Zheng Q. Li W. Zhang H. Gao X. Tan S. (2020). Optimizing synchronous extraction and antioxidant activity evaluation of polyphenols and polysaccharides from Ya'an Tibetan tea (*Camellia sinensis*). Food Sci. Nutr. 8, 489–499. 10.1002/fsn3.1331 31993173 PMC6977498

[B38] Zhong Y.-B. Kang Z.-P. Wang M.-X. Long J. Wang H.-Y. Huang J.-Q.. (2021). Curcumin ameliorated dextran sulfate sodium-induced colitis via regulating the homeostasis of DCs and Treg and improving the composition of the gut microbiota. J. Funct. Foods 86:104716. 10.1016/j.jff.2021.104716

[B39] Zhou D. D. Saimaiti A. Luo M. Huang S. Y. Xiong R. G. Shang A.. (2022). Fermentation with tea residues enhances antioxidant activities and polyphenol contents in Kombucha beverages. Antioxidants 11:155. 10.3390/antiox11010155 35052659 PMC8772747

[B40] Zhou F. Li Y. L. Zhang X. Wang K. B. Huang J. A. Liu Z. H.. (2021). Polyphenols from Fu brick tea reduce obesity via modulation of gut microbiota and gut microbiota-related intestinal oxidative stress and barrier function. J. Agric. Food Chem. 69, 14530–14543. 10.1021/acs.jafc.1c04553 34752089

[B41] Zhu M. Z. Li N. Zhou F. Ouyang J. Lu D. M. Xu W.. (2020). Microbial bioconversion of the chemical components in dark tea. Food Chem. 312:126043. 10.1016/j.foodchem.2019.126043 31896450

