# Supplementary material for: Bacillus licheniformis-based intensive fermentation of Tibetan tea improved its bioactive compounds and reinforced the intestinal barrier in mice
Source: Front Microbiol. 2024 Jun 12;15:1376757. doi: 10.3389/fmicb.2024.1376757 (PMC11199413; doi:10.3389/fmicb.2024.1376757)
Supplement: Supplementary file 1 [file Data_Sheet_1.docx]

Supplementary Material


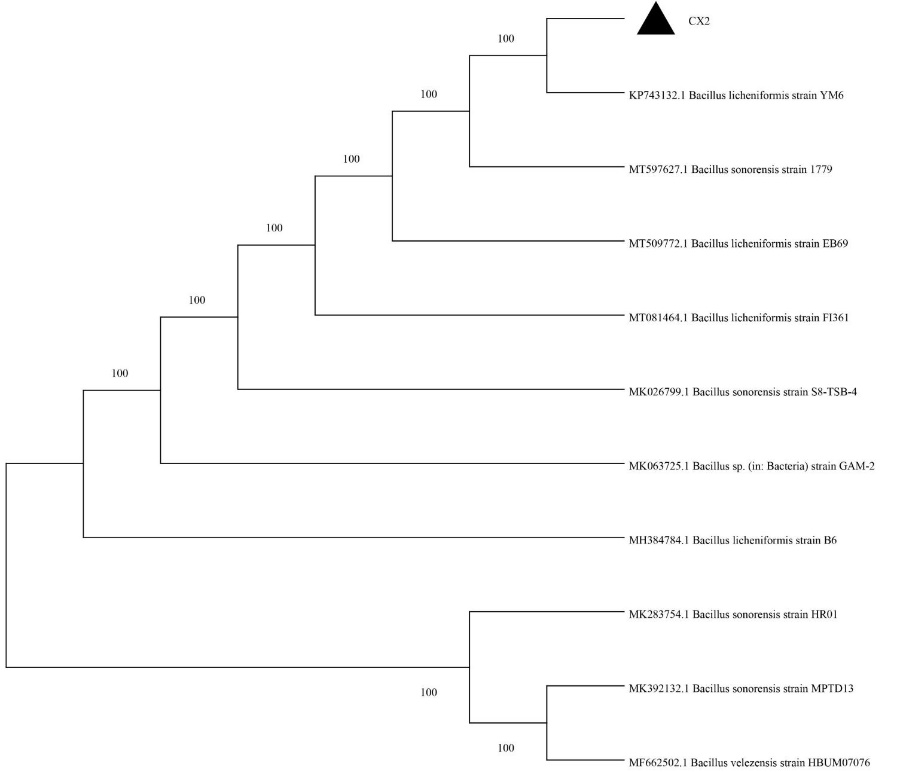


**Fig S.1 Phylogenetic tree constructed based on strain CX2**

**Table S.1 Differential compounds and their peak areas in Tibetan tea**

|  | C1 | C2 | C3 | BL1 | BL2 | BL3 |
| --- | --- | --- | --- | --- | --- | --- |
| Afzelechin-3-gallate | 409.9 | 454.399 | 438.03097 | 0 | 0 | 0 |
| Epiafzelechin-3-gallate | 419.9 | 472.395 | 508.22315 | 0 | 0 | 0 |
| Erucic acid | 4249 | 4036.55 | 3915.4535 | 0 | 0 | 0 |
| Mead acid | 0 | 0 | 0 | 201.4 | 232.47 | 280.256 |
| Epicatechin 3,5-digallate | 0 | 0 | 0 | 387.3 | 327.573 | 292.754 |
| 3,4-Dihyddroxy-benzoic acid | 0 | 0 | 0 | 418 | 492.18 | 386.36 |
| Protocatechuic acid | 0 | 0 | 0 | 418 | 492.18 | 430.54 |
| Epicatechin-3-O-(4'-O- methyl)-gallate | 82.9 | 195.736 | 121.96392 | 427.9 | 403.621 | 493.621 |
| Caffeic acid isomer | 0 | 0 | 0 | 401.4 | 458.33 | 479.856 |
| Paullinic acid | 1073 | 1161.27 | 1122.8827 | 675.6 | 695.868 | 682.356 |
| 2-Hydroxydiplopterol | 0 | 0 | 0 | 712.3 | 774.477 | 813.592 |
| p-Hydroxy ethyl cinnamate | 395 | 427.45 | 491.7245 | 860.1 | 813.997 | 896.203 |
| p-hydroxyacetophenone | 0 | 0 | 0 | 1003 | 1083.03 | 1153 |
| Naringin | 0 | 0 | 0 | 1058 | 1123.26 | 1204.32 |
| Feruloylquinic acid | 290.6 | 226.412 | 308.26848 | 1202 | 1238.06 | 1189.98 |
| L-Phenylalanine | 0 | 0 | 0 | 1255 | 1392.65 | 1267.55 |
| Naringenin-7-O-glucoside isomer B | 2546 | 2506 | 2581.46 | 1549 | 1495.47 | 1593.51 |
| Hydroxy linoleic acid | 3909 | 3899.61 | 3977.6022 | 1661 | 1594.56 | 1747.44 |
| Linoleic acid | 3939 | 3820.83 | 3985.4549 | 1681 | 1577.95 | 1644.39 |
| 3β-O-(8-Hydroxyoctanoyl)-12-oleanene | 979.9 | 1119.096 | 1039.47792 | 1765 | 1853.25 | 1707.35 |
| Dihydroquercetin | 0 | 0 | 0 | 2046 | 1984.62 | 2086.46 |
| Dihydroquercetin  isomer D | 0 | 0 | 0 | 2046 | 2148.3 | 1984.62 |
| Taxifolin | 0 | 0 | 0 | 2046 | 2107.38 | 1964.16 |
| Gallic acid | 0 | 0 | 0 | 2715 | 2827.35 | 2799.9 |
| 2,3,4-Trihydroxybenzoic acid | 0 | 0 | 0 | 2745 | 2625.2 | 2690.1 |
| 4-hydroxycinnamic acid | 0 | 0 | 0 | 2963 | 3161.15 | 3081.52 |
| p-Coumaric acid | 0 | 0 | 0 | 2963 | 3062.26 | 2874.11 |
| 5,7-Dihydroxycoumarin | 0 | 0 | 0 | 4959 | 3959 | 4038.18 |
| α-Spinasterol | 269.9 | 206.405 | 248.71285 | 4145 | 4020.65 | 4195 |
| α-Spinasterone | 1138 | 1026.62 | 1190.4186 | 4604 | 4373.8 | 4465.88 |
| 5-O-(3,4-Dimethoxycinnamoyl) quinic acid | 1854 | 1901.88 | 1970.5176 | 4688 | 4594.24 | 4875.52 |
| vanillic acid. | 0 | 0 | 0 | 5149 | 5046.02 | 5194 |
| Cinchonain-lb | 3008 | 3108 | 3188.32 | 5499 | 5549 | 5653.99 |
| Veratric acid | 0 | 0 | 0 | 5955 | 6252.75 | 6174.1 |
| Eicosadienoic acid | 0 | 0 | 0 | 7623 | 7851.69 | 7546.77 |
| β-Amyrin | 0 | 0 | 0 | 7944 | 8123.44 | 7785.12 |
| Caffeic acid | 0 | 0 | 0 | 8356 | 8272.44 | 8105.32 |
| Astragalin | 0 | 0 | 0 | 11040 | 10488 | 10929.6 |
| Astragalin isomer A | 0 | 0 | 0 | 11040 | 11592 | 11150.4 |
| Ricinoleic acid | 8964 | 8515.8 | 8175.168 | 12900 | 13029 | 13416 |
| 3-O-caffeoylquinic acid | 0 | 0 | 0 | 15210 | 14601.6 | 14753.7 |
| Chlorogenic acid isomer | 0 | 0 | 0 | 14601.6 | 15666.3 | 15210 |
| Chlorogenic acid | 0 | 0 | 0 | 15210 | 15362.1 | 15057.9 |
| Theogallin | 0 | 0 | 0 | 15310 | 15210 | 15463.1 |
| 3-O-galloylquinic acid | 0 | 0 | 0 | 15003.8 | 14697.6 | 15310 |
| (+)-Matairesinol | 0 | 0 | 0 | 17250 | 16905 | 15250 |
| (–)-Pinoresinol | 0 | 0 | 0 | 17077.5 | 17250 | 16905 |
| Teadenol | 0 | 0 | 0 | 17360 | 17012.8 | 17707.2 |
| Tea Polyphenols | 0 | 0 | 0 | 21920 | 21262.4 | 21481.6 |
| Phloroglucinol | 0 | 0 | 0 | 27120 | 27933.6 | 28204.8 |
| Pyrogallol | 0 | 0 | 0 | 27120 | 25764 | 27391.2 |
| β-Amyrone | 0 | 0 | 0 | 34160 | 32793.6 | 34843.2 |
| Icariside B5 | 0 | 0 | 0 | 35170 | 35873.4 | 33763.2 |
| Methyl gallate | 0 | 0 | 0 | 36850 | 38324 | 35376 |
| Vicenin-2 | 0 | 0 | 0 | 37790 | 37612.1 | 37412.1 |
| Biorobin | 0 | 0 | 0 | 37790 | 36656.3 | 37590 |
| Nicotiflorin | 0 | 0 | 0 | 37790 | 37412.1 | 37690 |
| Myricetin-3-O-β-d-glucopyranoside | 0 | 0 | 0 | 43550 | 42243.5 | 45392 |
| Hyperoside | 0 | 0 | 0 | 44210 | 44652.1 | 43767.9 |
| Quercetin-3-O-β-d-glucopyranoside | 0 | 0 | 0 | 44210 | 44510 | 45094.2 |
| Quercetin-3-O-β-d-galactopyranoside | 0 | 0 | 0 | 44210 | 44652.1 | 45978.4 |
| Kaempferol | 0 | 0 | 0 | 44730 | 43835.4 | 46071.9 |
| Kaempferol isomer A | 0 | 0 | 0 | 44730 | 45177.3 | 43835.4 |
| Luteolin | 0 | 0 | 0 | 44730 | 45624.6 | 45282.7 |
| Vitexin | 0 | 0 | 0 | 46370 | 48688.5 | 47761.1 |
| Kaempferol-3-O-α-l-rhamnopyranoside | 0 | 0 | 0 | 46370 | 45442.6 | 48224.8 |
| Kaempferol 3,7-di-O-rhamnoside | 0 | 0 | 0 | 57490 | 58639.8 | 56340.2 |
| Apigenin-6-glucoside-8-arabinoside | 0 | 0 | 0 | 57560 | 55833.2 | 58711.2 |
| Isoschaftoside | 0 | 0 | 0 | 57360 | 57560 | 56408.8 |
| Myricetin | 0 | 0 | 0 | 80570 | 84598.5 | 79764.3 |
| Myricetin isomer | 0 | 0 | 0 | 80570 | 76541.5 | 78152.9 |
| Quercetin | 235.9 | 208.259 | 298.259 | 81120 | 84364.8 | 84264.8 |
| Quercetin isomer A | 235.9 | 202.977 | 257.83654 | 81120 | 84364.8 | 80308.8 |
| Quercetin isomer B | 235.9 | 294.105 | 198.5871 | 81120 | 83553.6 | 79497.6 |
| Quercetin isomer C | 255.9 | 326.464 | 219.67008 | 81120 | 77064 | 78686.4 |
| (-)-EC | 0 | 0 | 0 | 88060 | 91582.4 | 87179.4 |
| Catechin | 0 | 0 | 0 | 88060 | 83657 | 88940.6 |
| Epicatechin | 0 | 0 | 0 | 88060 | 86298.8 | 84537.6 |
| (-)-EGC | 0 | 0 | 0 | 133700 | 140385 | 131026 |
| (-)-Epicatechin gallate/ECG | 761.7 | 784.551 | 716.01447 | 266700 | 256032 | 272034 |
| tiglylglycine | 0 | 0 | 0 | 271700 | 282568 | 279851 |
| L-Theanine | 0 | 0 | 0 | 401300 | 389261 | 389561 |
| 4-hydroxybenzoic acid | 1577 | 1509.69 | 1468.5024 | 490700 | 485793 | 475979 |
| Salicylic acid | 1577 | 1605.85 | 1672.4085 | 490700 | 480886 | 490800 |
| Epigallocatechin gallate/EGCG | 1258 | 1146.42 | 1069.3484 | 517700 | 522877 | 512523 |
| Dihydrokaempferol | 673.7 | 750.648 | 602.62208 | 538000 | 559520 | 532620 |
| Caffeine | 172100 | 168658 | 163598.26 | 8246000 | 7998620 | 8248000 |

**
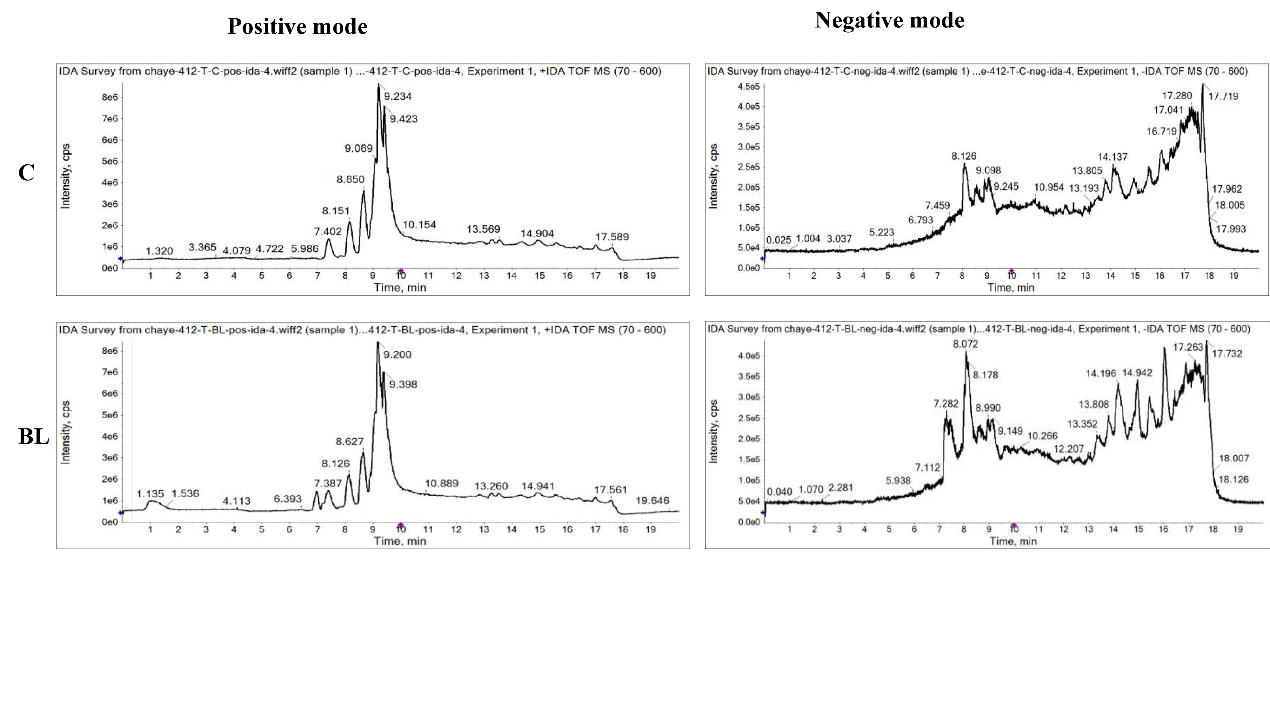
**

**Fig S.2 Total ion chromatogram (TIC) for** **Tibetan tea** **by fermentation with Bacillus licheniformis (C) and non-fermented (BL)**
